# Treatment options for acute myeloid leukemia patients aged <60 years

**DOI:** 10.3389/fonc.2022.897220

**Published:** 2022-10-05

**Authors:** Giuseppe Visani, Martina Chiarucci, Sara Paolasini, Federica Loscocco, Alessandro Isidori

**Affiliations:** Hematology and Stem Cell Transplant Center, Azienda Ospedaliera "Ospedali Riuniti Marche Nord" (AORMN), Pesaro, Italy

**Keywords:** AML - acute myeloid leukemia, target therapy, induction, allogeneic stem cell transplantation, young

## Abstract

Treatment of acute myeloid leukemia (AML) has changed over the last few years, after the discovery of new drugs selectively targeting AML blasts. Although 3/7 remains the standard of care for most AML patients, several new targeted agents (such as FLT3 inhibitors, CPX-351, gemtuzumab ozogamicin, BCL-2 inhibitor, and oral azacitidine), either as single agents or combined with standard chemotherapy, are approaching clinical practice, starting a new era in AML management. Moreover, emerging evidence has demonstrated that high-risk AML patients might benefit from both allogeneic stem cell transplant and maintenance therapy, providing new opportunities, as well as new challenges, for treating clinicians. In this review, we summarize available data on first-line therapy in young AML patients focusing on targeted therapies, integrating established practice with new evidence, in the effort to outline the contours of a new therapeutic paradigm, that of a “total therapy”, which goes beyond obtaining complete remission.

## Introduction

Acute myeloid leukemia (AML) is a malignant disorder of the hematopoietic system mostly diagnosed in the elderly population. However, it can occur at any age. Previously incurable, only 35% to 40% of younger patients (aged <60 years) and 5% to 15% of older patients are alive and disease-free at 5 years (1). Historically, long-term survival without allogeneic hematopoietic stem cell transplantation (allo-HSCT) has been extremely poor (2). Results from 2,551 adults with AML who received intensive chemotherapy on Cancer and Leukemia Group B (CALGB) trials before the targeted therapy era, without undergoing allo-HSCT in first remission, showed 10-year disease-free survival (DFS) in only 15% of younger patients and <2% of older patients (3). This poor outcome is in part due to a myriad of chromosomal alterations and gene mutations, which are frequently found in AML blasts, thereby promoting a clinically heterogeneous group of diseases, which share the common features of drug resistance and high relapse rate (2). Despite steady progress in the understanding of AML biology and novel technologies to better characterize the biology of the disease, such as multicolor flow cytometry, droplet digital polymerase chain reaction (ddPCR), and next-generation sequencing (NGS), the treatment paradigm has not drastically changed during the last 40 years. Therefore, there are still relevant unmet needs to improve survival and quality of life for most AML patients by optimizing the combination of novel therapies with conventional agents, including innovative approaches to allo-HSCT. Here, we will discuss current therapeutic approaches in young AML patients and future prospects with a focus on promising drugs in development.

## General considerations

Treatment of AML depends on several prognostic factors including age, overall health status, and presence of genetic or chromosomal abnormalities. Thus, there is a common trend to better characterize AML subtypes at diagnosis, to stratify tailored therapies earlier in the treatment course. AML has often been assumed to require urgent treatment; however, data from a cohort of 599 patients reported that time from diagnosis to initiation of intense treatment (TDT) had no effect on survival even in patients presenting with white blood cells (WBCs) > 50,000/μl or age > 60 years (4). Röllig et al. recently confirmed these results analyzing data from a registry of 2,200 patients, suggesting that TDT is not related to response or survival, neither in younger nor in older patients (5).

The choice between conventional and investigational therapy is guided by ELN 2017 recommendations (2), in which patients are divided into “favorable”, “intermediate”, and “adverse” groups. Recently, the German Group has validated this stratification for remission, survival, and relapse-free survival (6). In particular, patients with TP53 mutations and/or complex cytogenetics, which are highly associated with each other, showed a particularly dismal outcome and should be considered a distinct “very adverse” group, which may benefit most by enrolment in a clinical trial. Using this refined classification, complete remission (CR) rates for the very favorable [patients with inv(16)/t(16;16) or biallelic CEBPA mutations], favorable, intermediate, adverse, and very adverse groups were 77%, 71%, 66%, 44%, and 27%, respectively, and estimated OS rates at 5 years were 70%, 50%, 31%, 14%, and 0%, respectively (6). It is important to keep in mind that risk classification systems must always be interpreted in conjunction with treatment regimens, which may change over time. In this view, neither ELN2017 nor its modifications incorporate data from patients treated with new drugs recently approved. Finally, prediction of long-term outcome based on pre-treatment disease-related factors alone has a major bias, since patient-related factors are not included in the ELN categories. Criteria for unfitness are, in most trials, age either ≥ 65/70/75 years, performance status, or comorbidities, all precluding the use of intensive induction. As known, “geriatric assessment” has been shown to refine the prognostic effect of age, which should not be the only determinant of fitness for intensive induction, as reported in ELN 2017 (2). A panel of experts from the Italian Society of Hematology (SIE) has proposed a new definition of unfitness to intensive and non-intensive chemotherapy (7), using an analytic hierarchy process-based consensus process. The definition of unfitness to intensive therapy should require the fulfillment of at least one of nine criteria (**Table 1**).

**Table 1:** Conceptual and operation criteria to define AML patients’ unfitness to intensive chemotherapy.

Conceptual criteria	Operation criteria
**Advanced age (over 75 years)**	An age older than 75 years
**Severe cardiac comorbidity**	Congestive heart failure or documented cardiomyopathy with an EF ≤ 50%
**Severe pulmonary comorbidity**	Documented pulmonary disease with DLCO ≤ 65% or FEV1 ≤ 65%, or dyspnea at rest or requiring oxygen, or any pleural neoplasm or uncontrolled lung neoplasm
**Severe renal comorbidity**	On dialysis and age older than 60 years or uncontrolled renal carcinoma
**Severe hepatic comorbidity**	Liver cirrhosis Child B or C, or documented liver disease with marked elevation of transaminases (>3 times normal values) and an age older than 60 years, or any biliary tree carcinoma or uncontrolled liver carcinoma or acute viral hepatitis
**Active infection resistant to anti-infective therapy**	Active infection resistant to anti-infective therapy
**Cognitive impairment**	Current mental illness requiring psychiatric hospitalization, institutionalization or intensive outpatient management, or current cognitive status that produces dependence (as confirmed by the specialist) not controlled by the caregiver
**Low performance status (ECOG functional scale)**	ECOG performance status ≥3 not related to leukemia
**Any other comorbidity that the physician judges to be incompatible with conventional intensive chemotherapy**	Any other comorbidity that the physician judges to be incompatible with conventional intensive chemotherapy

AML, acute myeloid leukemia; DLCO, diffusing capacity of the lungs for carbon monoxide; ECOG, Eastern Cooperative Oncology Group; EF, ejection fraction; FEV1, forced expiratory volume in 1 s.

Moreover, analyses of measurable residual disease (MRD) during and after treatment by flow cytometry [with a threshold set at 0.1%, the amount of residual leukemic cells (2)], quantitative PCR, or NGS have emerged as novel tools to assess response to therapy and to guide the post-remission strategy. Different methods have specific indications and require highly specialized expertise. The ELN Working Party consensus document on MRD in AML (2) indicates that molecular assessment for NPM1 mutations, RUNX1-RUNX1T1, CBFB-MYH11, and PML-RARA fusion transcripts should be performed at diagnosis, at least after two cycles of induction/consolidation therapy, and every 3 months, for 24 months after the end of treatment. Monitoring of NPM1-mutated transcripts may be more informative when performed in PB as compared to BM. In general, obtaining an MRD-negative CR is associated with longer remissions, DFS, and OS, independently from the intensity of chemotherapy and allo-HSCT. Accordingly, as recognized by the ELN2017 panel (1), the goal of induction therapy should be CR with MRD negativity in all patients, independently from the type of treatment. In addition to MRD assessment, the detection of small subclones by NGS is important to evaluate clonal evolution during the disease course (even though its current error rates set the sensitivity level at about 1%). Regarding therapeutic intervention in patients with an MRD positivity after allo-HCT, different strategies might be pursued. Fast tapering of immunosuppressive treatment, donor lymphocyte infusion, hypomethylating agents, or FLT-3 inhibitors are valid options, and might be used alone or in combination with each other (8).

## First-line treatment

The mainstay treatment for AML in the fit population is based on an induction chemotherapy with cytarabine plus an anthracycline, with or without a purine analogue, followed by two to four cycles of consolidation chemotherapy and/or allo-HSCT, to eliminate residual leukemic cells (2). This approach achieves CR in 60%–70% of patients aged <60 years, with a lower remission rate and a dismal outcome in patients with adverse risk disease.

Intensive induction therapy is based on 7 days cytarabine with 3 days anthracycline (i.e., 7 + 3). Several trials have been done to improve the outcome of “7 + 3”: adding a third drug, and increasing the dose of cytarabine and daunorubicin.

The SWOG trial 1203 randomized 754 adults aged < 60 years among 7 + 3 (with a daunorubicin dose of 90 mg/m^2^), idarubicin + cytarabine 1.5 g/m^2^ daily by continuous infusion × 4 days (IA) and IA + vorinostat (9). CR rates were 75%–79%, and there were no differences in EFS, RFS, or survival among the three arms, neither in patients with NPM1, FLT3, or CEBPA mutations or intermediate or adverse cytogenetics. In patients with favorable cytogenetics, outcomes were significantly better with 7 + 3 than with IA or IA + V; however, different doses of cytarabine were administered during post-remission therapy, and this may have led to confusing results (9).

The NCRI/MRC group (AML15 trial) randomized 3,106 AML patients in two comparison arms, with the aim to compare cytarabine, daunorubicin, etoposide (ADE) with daunorubicin and cytarabine (DA), fludarabine, cytarabine, granulocyte colony-stimulating factor (G-CSF), and idarubicin (FLAG-Ida). They observed reduced relapse rates using FLAG-Ida instead of ADE and DA, but no survival benefits due to higher treatment-related mortality after obtaining CR, in the former group (10).

The randomized phase 2 trial on alvocidib, cytarabine, and mitoxantrone hydrochloride (FLAM) compared to cytarabine and daunorubicin hydrochloride (3 + 7) was conducted on 165 treating patients with newly diagnosed AML. The study showed no significant differences in overall survival (OS) between FLAM and 3 + 7, despite significantly higher CR rates with FLAM. The OS appeared to be better in patients <50 years old, compared with those ≥50 years old, without significant differences (11, 12).

A phase 1/2 trial of G-CSF, cladribine, cytarabine, and dose-escalated mitoxantrone (G-CLAM), conducted on 199 patients with newly diagnosed or relapsed/refractory AML or high-risk myelodysplastic syndromes, showed higher rates of CR/CRi and higher MRD-negative CR (measured by multiparameter flow cytometry [MFC]) than standard “7 + 3” therapy in fit patients with newly diagnosed AML or other high-grade myeloid neoplasm with ≥10% blasts (HG-MN) (13).

Results of E1900 reported in 2009 (14) showed a benefit of anthracycline intensification [45 (standard dose) versus 90 (high dose) mg/m^2^ daunorubicin for 3 days in combination with cytarabine] in younger patients, but not in patients with adverse cytogenetics, FLT3-ITD, or aged 50 years or older. In 2016, the updated results of the same trial reported broader benefit in high-risk patients, both in unfavorable cytogenetics and in FLT3-ITD mutant AML, with a 10% advantage; the FLT3-ITD positive patients received a higher dose of daunorubicin (15).

Another study that evaluated different doses of daunorubicin, in the same period, was the HOVON trial (16), although this study enrolled only patients aged ≥60 years, eligible for intensive chemotherapy. DFS and OS for AML patients treated with high-dose daunorubicin were not superior to standard dose in the whole population. However, there was a benefit of survival in patients aged 60–65 years.

Another randomized trial, which compared standard versus high-dose daunorubicin induction in 383 young adults with AML, was performed in Korea and published in 2011 (17). High-dose daunorubicin produced a statistically significant higher DFS and OS after a median follow-up of 52.6 months. The survival benefits of high-dose daunorubicin therapy were more prominent in patients with intermediate-risk cytogenetics, and toxicities were similar in the two arms.

Finally, patients with FLT3 ITD (*n* = 200) appear to have fewer relapses leading to longer survival when treated with the 90 mg/m^2^ dose of daunorubicin (18). In this setting, Luskin et al. (15) showed a benefit in a significant subgroup of older patients aged 50 to 60 years with FLT3-ITD or NPM1 mutations. In particular, NPM1 mutant patients receiving intensified daunorubicin had a remarkable increase in median OS (75.9 vs. 16.9 months) and a >20% increase in 4-year OS (52% vs. 29%) (15).

Current evidence suggests that the dose of daunorubicin should not be less than 60 mg/m^2^ (2).

Although 3/7 remains the standard of care for most AML patients, several new targeted agents, already approved or under investigation, either as single agents or combined with standard chemotherapy, are approaching clinical practice, starting a new era in AML management. Since 2017, the US Food and Drug Administration (FDA, **Table 2**) have approved nine new drugs, and some of these have already been incorporated in clinical practice.

**Table 2:** Recent FDA approvals in newly diagnosed AML.

Drug or regimen	FDA approval indication
Midostaurin	FLT3^MUT^ AML
CPX-351 (liposomal daunorubicin HCl and cytarabine)	tAML, AML MRC
Gemtuzumab Ozogamicin	Newly diagnosed adults with CD33+ AML with IC
Glasdegib + LDAC	Newly diagnosed AML >75 years or unfit for IC
Venetoclax +HMA	Newly diagnosed AML >75 years or unfit*
Venetoclax + LDAC	Newly diagnosed >75 years or unfit*
Ivosidenib	Newly diagnosed >75 years or unfit* with IDH1^MUT^ RR AML with IDH1^MUT^
Enasidenib mesylate	RR AML IDH2^MUT^
Oral azacytidine	Post-IC Maintenance

AML MRC, and AML with myelodysplasia-related changes; HMA, hypomethylating agent; IC, intensive chemotherapy; LDAC, low-dose cytarabine; t-AML, therapy-related AML; RR, relapsed/refractory. *Adult patients with newly diagnosed AML who are >75 years old or who have comorbidities that preclude use of intensive induction chemotherapy.

Finally, it is broadly accepted that post-remission therapy is needed to prevent relapse. Allo-HSCT reduces the risk of relapse, with its graft vs. leukemia (GVL) effect. Nonetheless, allo-HSCT is affected by a significant non-relapse mortality and morbidity, due to acute and chronic graft vs. host disease (GVHD). The general recommendation is to offer allo-HSCT to all patients with an ELN2017 risk either intermediate or adverse. In contrast, the risk of relapse in the ELN 2017 favorable subgroup is low, and does not justify the use of allo-HSCT in this setting. However, patients with MRD-positive CR should be candidates for frontline allo-HSCT, regardless of the initial risk group, in order to improve OS and DFS. In fact, MRD positivity after the second course of chemotherapy significantly affects OS in patients with favorable and standard risk AML, as reported by Freeman et al. (19), much more than in high-risk patients. Moreover, the presence of NPM1-mutated transcripts after the second cycle of chemotherapy in the peripheral blood of patients in CR was the only significant adverse prognostic factor in the UK NCRI AML 17 trial (20). In summary, allo-HSCT remains the best post-remission treatment option for the vast majority of AML patients. It is still debated whether maintenance after allo-HCT is warranted and if it should be reserved only to high-risk patients, or to the entire population submitted to allo-HCT.

### FLT3 inhibitors

FMS-like tyrosine kinase 3 (FLT3) gene mutations are present in approximately 30%–35% of all AML patients. Within this subgroup, 25% of AML patients show internal tandem duplications (ITDs), whereas 5%–8% present a mutation in the tyrosine kinase domain (TKD) (21). The presence of a high FLT3-ITD allelic ratio of the TKD mutation confers a poor prognosis (2). FLT3-mutated AML is the paradigm for therapies combining molecularly targeted agents with standard intensive chemotherapy.

First-generation FLT3 inhibitors were not specifically designed to target the FLT3 receptor. These drugs are nonspecific, multikinase inhibitors, with additional activity against other targets such as c-Kit, platelet-derived growth factor receptor (PDGFR), and vascular endothelial growth factor receptor (VEGFR). This class of drugs include lestaurtinib, sunitinib, sorafenib, ponatinib, and midostaurin. In 2017, based on the randomized “RATIFY” trial, the FDA approved 3 + 7 plus midostaurin for adults aged <60 years with FLT3-ITD or TKD mutation, regardless of the allelic ratio. In this global, randomized, placebo-controlled phase 3 trial of 717 patients, the addition of midostaurin to induction and consolidation chemotherapy resulted in superior survival rates compared to chemotherapy alone (7.2% difference in 4-year OS) (22). The superiority of midostaurin was independent of allo-HCT, with best results seen in patients receiving allo-HCT in CR1. Moreover, there was a trend toward better OS in all FLT3-mutation subtypes (TKD, ITD with low allelic ratio, and ITD with high allelic ratio) in the midostaurin arm (17). Another endpoint of the trial was to test the efficacy of 12-month maintenance with midostaurin, but only in patients not submitted to allo-HCT. Among 717 enrolled patients, only 174 began maintenance therapy, and only 104 completed the 12-month schedule. In the unplanned *post-hoc* subset analysis of the CALGB 10603/RATIFY trial reported by Larson et al. (23), there was no difference in DFS between the two arms (HR = 1.4 [95% CI, 0.63–3.3]; *p* = 0.38) from the end of maintenance. Moreover, midostaurin did not produce an improvement in OS from the start of maintenance (HR = 0.96 for M [95% CI, 0.58–1.59]; *p* = 0.86). Accordingly, midostaurin was licensed for the treatment of adult patients with newly diagnosed AML who are FLT3+ in combination with standard cytarabine and daunorubicin induction and cytarabine consolidation, but not as a single agent for maintenance therapy.

In order to answer some of the unanswered questions left by the RATIFY trial, the German–Austrian AML Study Group (AMLSG) designed the 16-10 trial. This was a phase 2, open-label, single-arm study that enrolled patients aged 18–70, designed to test the role of midostaurin during induction and consolidation, in association with chemotherapy, and as a maintenance (12 months) after allo-HCT (24). The trial enrolled 284 patients with FLT3-ITD only; 134 patients received allo-HCT during the first complete remission (CR1), 75 of whom received midostaurin maintenance. Patients started midostaurin after a median time of 71 days post-allo-HCT. The median duration of maintenance was 9 months, and toxicities were mainly gastrointestinal (70%) and infections (51%), but were low in grade and manageable. The 16-10 trial showed a statistically significant advantage for midostaurin maintenance over no maintenance for both EFS (*p* = 0.01, HR 2.51) and OS (*p* = 0.02, HR 2.64) (24).

Recently, the RADIUS trial tested, in a randomized fashion, the efficacy of 12-month maintenance with midostaurin after allo-HCT in 60 FLT3-ITD+ AML patients. Thirty patients received midostaurin, and 30 received standard-of-care therapy. However, only 16 patients in the midostaurin arm and 14 in the placebo arm completed the 12 months of treatment. The primary outcome, RFS, was comparable in the two arms. There were no differences in 24-month OS (25). A positive note regarded toxicity, intended as serious adverse events and GVHD (any grade) during maintenance, because there was no significant difference between midostaurin and placebo. Given the small sample size of the trial, it is not possible to draw a definitive conclusion on the role of midostaurin after allo-HCT.

Despite being not licensed for the treatment of AML, sorafenib has been one of the first and most widely used FLT3 inhibitors. It is an orally administered multikinase inhibitor with potent inhibitory activity against the Raf kinase and MAPK signaling pathway and FLT3, c-Kit, VEGFR, RET, and PDGFR. This drug was evaluated in many clinical trials in AML patients, both in combination with induction chemotherapy and for maintenance. Ravandi et al. reported a high response rate in patients with previously untreated AML who received a combination of sorafenib, cytarabine, and idarubicin (CR with incomplete recovery rates were 95%) (26).

In the SORAML randomized trial, sorafenib combined with standard induction chemotherapy significantly prolonged EFS (26 months) and RFS (63 months) as compared with placebo plus chemotherapy (9 and 22 months, respectively) in younger patients (≤60 years). Notably, only 17% of these patients had FLT3-mutated AML (27), and no significant difference was observed for patients with FLT3-ITD. The SORAML trial indicated, for the first time, the role of a maintenance therapy in young AML patients, independently from molecular aberrations, in addition to standard chemotherapy.

Additionally, in the context of post-transplant maintenance, results of the randomized phase 2 SORMAIN study (28) indicated that sorafenib reduces the risk of relapse (2-year RFS was 85% in the sorafenib group compared with 53.3% in the placebo group) and death. However, although well tolerated, sorafenib maintenance was associated with higher incidence of acute and chronic GvHD (≥3 grade: 76.8% in the sorafenib arm vs. 59.8% in the placebo group). More recently, Bazarbachi et al. reported the results of the European Group for Blood and Marrow Transplantation (EBMT) registry-based study of 62/462 patients with FLT3-mutated AML (FLT3-ITD-95%) who received posttransplant sorafenib either as a prophylactic (*n* = 19), as pre-emptive therapy (*n* = 9), or as treatment for relapse (*n* = 34) (29). On multivariate analysis, maintenance with sorafenib significantly improved leukemia-free survival (LFS) (hazard ratio [HR] = 0.35), OS (HR = 0.36), and graft-versus-host disease-free RFS (HR = 0.44).

On the other hand, second-generation inhibitors, such as gilteritinib, quizartinib, and crenolanib, were designed to selectively and potently inhibit the FLT3 receptor, and presumably have an improved tolerability at the concentrations necessary to fully inhibit FLT3 *in vivo*. Many early-phase trials combining the more potent next-generation FLT3-TKIs with 7 + 3 induction chemotherapy in the frontline setting have been reported recently with meaningfully high response rates. Gilteritinib has demonstrated the potent and selective inhibitory activity of FLT3-ITD and FLT3-TKD mutations, with additional inhibitory activity against EML4-ALK and Axl. In accordance with the final results of the phase 3 ADMIRAL study (30), in 2018, the FDA approved gilteritinib for FLT3-mutated, relapsed/refractory disease. In 2018, Pratz et al. reported the updated results of a phase 1/2 study of gilteritinib combined with 7 + 3 and HIDAC consolidation in 62 unselected AML patients. The CRc rate was 100% in FLT3-mutated patients, with a median DFS of 297 days (31).

Currently, numerous clinical trials of gilteritinib are underway to evaluate its role in various settings such as first-line, rescue, consolidation, or maintenance. In this last setting, the BMT-CTN 1506 study is ongoing to establish whether there is a benefit of FLT3 inhibition in the post-HCT setting and, if so, in which patients. This study is a randomized, double-blind, placebo-controlled, phase 3 trial conducted on 346 AML patients with FLT3-ITD mutation, randomly assigned in a double-blinded fashion to placebo or gilteritinib (32).

Crenolanib is another potent FLT3 inhibitor with activity against both FLT3-ITD and FLT3-TKD with additional inhibitory activity of PDGFR and c-Kit. Additionally, crenolanib is a potent inhibitor of the mutant FLT3-D835, which is one of the main mechanisms of resistance to FLT3 inhibitors. The addition of crenolanib to standard chemotherapy has been assessed in 29 young patients (<60 years) with newly diagnosed FLT3-mutated AML included in a phase 2 trial. After 20.8 months of median follow-up, CR was achieved in 72% after one cycle of induction, 81% of patients were alive, and median overall survival, event-free survival, and cumulative incidence of relapse had not been reached (33, 34). The randomized phase 3 trial is currently evaluating the addition of crenolanib vs. midostaurin to standard chemotherapy in newly diagnosed FLT3 mutated AML patients, and has been designed to match the same inclusion criteria as those of the RATIFY trial.

In summary, midostaurin is the only approved FLT3 inhibitor in combination with induction chemotherapy in newly diagnosed FLT3-mutated AML and it can be used regardless of FLT3 mutation settings (ITD, TKD, ITD with NPM1 mutation, FLT3-ITD low, and ITD with poor prognostic driver mutations). However, based on the very high response rate achieved with next-generation FLT3 inhibitors (80%–90%), two phase 3 randomized trials are ongoing and investigating frontline quizartinib/crenolanib + 7 + 3 chemotherapy (NCT02668653 and NCT03258931 respectively) (**Table 3**). Recent data confirmed that allo-HCT remains the best consolidation therapy for AML patients with FLT3-ITD pertaining to the high-risk ELN category, and should be performed as soon as possible in CR1 regardless of the use of FLT3 inhibitors. It is important to remember that patients with NPM1mut/FLT3-ITD low (<0.50 AR) included in the intermediate-risk AML category should be carefully considered for allo-HCT as first-line treatment, given their more favorable outcome with respect to high-risk ELN (36–38).

**Table 3:** FLT3 inhibitors combined with intensive chemotherapy in frontline AML therapy: clinical trials.

FLT3 inhibitor + chemotherapy	Trial design	Enrolled/goal	Primary results to date
Midostaurin vs. placebo plus 7 + 3 (cytarabine + daunorubicin) (22)	Phase 3	717 patients	CR/CRi: Midostaurin 54% vs. placebo 59% (*p* = NS) 4-year OS: Mido 51.4% vs. 44.3% (7.1% difference)
Midostaurin plus 7 + 3 (cytarabine + daunorubicin or idarubicin) and maintenance NCT03379727	Phase 3b	300 patients	Completed accrual; Results pending
Quizartinib plus 7 + 3 (35)	Phase 1	19 patients	CR/CRi = 84%; well tolerated
Quizartinib vs. placebo plus 7 + 3 (cytarabine + daunorubicin) NCT02668653	Phase 3	539 patients	Completed accrual; Results pending
Crenolanib plus 7 + 3 (cytarabine + daunorubicin/idarubicin) (33)	Phase 2	29 patients	CR/CRi = 72%; 2-year OS not reached
Crenolanib vs. midostaurin plus 7 + 3 (cytarabine + daunorubicin) NCT03258931	Phase 3	510 patients	Currently accruing
Gilteritinib plus 7 + 3 (cytarabine + idarubicin or daunorubicin) (31)	Phase 1	70 patients	CR/CRi = 93%

## BCL2 inhibitors/venetoclax

Recently published phase 3 studies have established a new standard-of-care therapy in patients not eligible for intensive chemotherapy in light of survival benefit for patients treated with azacitidine plus venetoclax or LDAC plus venetoclax (39, 40). Where and when targetable mutations are found, it is expected that therapy may be optimized with the molecular-targeted doublet (i.e., substitution of HMA or LDAC for a molecularly targeted therapy) or triplet (the addition of a molecular-targeted therapy to a venetoclax backbone) with appropriate schedule modifications. These studies have also paved the way for other uses of venetoclax. In particular, in the setting of fit, younger patients, the combination of venetoclax with intensive chemotherapy and the role in the maintenance phase of therapy for patients in CR should be investigated. Early-phase trials have evaluated the combination of venetoclax with intensive chemotherapy and set the safety dose of venetoclax at 200 mg daily for 4 days in the younger population (41).

The MD Anderson group recently reported the interim results of a phase 1b/2 trial with the combination FLAG-IDA plus venetoclax in a mixed AML population (treatment naïve and R/R patients) (42). Twenty-nine newly diagnosed AML patients were enrolled. As expected, the most important grade III–IV toxicities were infections (febrile neutropenia 50%, bacteremia 35%, pneumonia 28%, and sepsis 12%). This combination induced a very high rate or MRD-negative composite CR (96%) in the newly diagnosed setting. Allo-HCT was performed in 69%, and 94% of patients were alive 1 year after the transplant. Even if the results are appealing, especially if we consider the high rate of MRD-negative remissions, numbers are very small and the median follow-up is too short to draw any significant conclusion. Patients with TP53 mutations continued to have poor outcomes despite treatment with this combination, and survival in TP53-mutated was significantly inferior to that of wild-type patients (43). Furthermore, a recent trial confirmed the safety and efficacy of the combination of venetoclax (400 mg *per os* on days 2–8) with the intensive CLIA regimen (cladribine, cytarabine, and idarubicin) in newly diagnosed AML. Fifty patients aged 18–65 years were enrolled; 47/50 (94%) achieved a remission, and only 1 patient died of toxicity during induction. Again, the combination induced a high rate of MRD-negative CR (82%), and infections were the most common grade III–IV adverse event reported. Notably, two patients with FLT-3 positive AML died due to infectious complications while on treatment with CLIA-VEN and FLT-3 inhibitors. Drug–drug interaction between venetoclax, FLT-3 inhibitors, and azoles, which are all metabolized *via* CYP3A, should be deeply investigated in order to avoid possible unexpected toxicities in this population of AML patients. Twelve-month EFS and OS are promising, but also in this case, we need to wait for more data (and more mature) in order to correctly weigh the impact of the combo in newly diagnosed AML (44).

Notably, the US investigators recently started a prospective, multicenter, double-blind, randomized, placebo-controlled phase 3 clinical study (NCT04628026) exploring the role venetoclax plus intensive chemotherapy in newly diagnosed patients with AML. Different dosages of venetoclax (100, 200, and 300) will also be tested in the trial, in order to clarify the role of bcl-2 inhibition in combination with intensive chemotherapy (**Table 4**).

**Table 4:** Bcl2 inhibitors/Venetoclax + intensive chemotherapy in AML young and fit patients.

Venetoclax + chemotherapy	Trial design	Enrolled/goal	Primary results to date
Venetoclax plus FLAG-IDA (NCT03214562)^36^	Phase 1b/2	116 patients	Recruiting. Results on 29 patients enrolled: CR MRD negative = 96%; allo-HCT = 69%; alive 1 year after transplant = 94%
Venetoclax plus CLIA (NCT02115295)^38^	Phase 2	458 patients	Recruiting. Results on 50 patients enrolled: CR MRD negative = 82%
Venetoclax plus 7 + 3 (NCT05356169)	Phase 2/3	300 patients	Not yet recruiting
Venetoclax and azacitidine for non-elderly adult patients (NCT03573024)	Phase 2	36 patients	Recruiting
Venetoclax plus intensive chemotherapy (7 + 3) (NCT04628026)	Phase 3	650 patients	Recruiting

Currently, too many questions remain unanswered: the dosage of venetoclax, the duration of therapy, the characteristics of the patients, both clinical and biological, and the lack of a randomized trial still do not allow us to recommend its use outside from clinical trials, in the younger population fit for intensive chemotherapy.

## Monoclonal antibodies

Since the early 1980s, attempts to use antigen-targeted immunotherapy to selectively kill AML cells resulted in the development of monoclonal antibodies (mAbs). However, none of them have demonstrated a significant efficacy to be incorporated into standard of care. Nonetheless, recent advancements in antibody engineering have attracted a lot of interest in antibody-based therapies in AML. Reinforcing mAbs by investigating agents with novel targets or mechanisms, as well as combination strategies, hold a promise to make a great progress in this field.

CD33 (SIGLEC-3) is a member of the sialic acid-binding immunoglobulin-like lectin (Siglec) family, which has been the most exploited target in AML treatment due to its expression on at least one subset of leukemic blasts in almost all patients. CD33 is highly expressed in acute promyelocytic leukemia (APL), NPM1-mutated AML, and FLT3/ITD mutated AML, whereas expression is usually low in leukemias with core-binding factor translocations (45).

Gemtuzumab ozogamicin (GO) is a highly potent antitumor antibiotic disulfide derivative of calicheamicin-γ1 conjugated to a recombinant humanized antibody (IgG4) against CD33. GO targets CD33, allowing the fast release of the toxic part in the tumor cell lysosomes (46). Calicheamicin binds to the minor groove of DNA and triggers single- and double-stranded breaks, inducing cell death through mitochondrial pathways and caspase activation. Single-drug activity in three uncontrolled phase 2 studies resulted in accelerated regulatory approval of GO in 2000 by FDA for patients with CD33-positive AML in first relapse (47). The preliminary data from phase 3 trial SWOG S0106 were associated with a significantly higher risk of fatal adverse events during the induction phase, which led to early trial termination and the decision to withdraw the drug in 2010 (48). Four other randomized trials investigated whether adding GO to the first cycle of intensive chemotherapy of adults with newly diagnosed AML improved outcomes: MRC/NCRI AML-15 (49), AML16 (50), ALFA-0701 (51), and GOELAMS AML 2006 IR (52). Despite the heterogeneity between the studies, adding GO resulted in significantly improved survival in all studies except S0106 (48), which, unlike the other studies, used a lower daunorubicin dose (45 mg/m^2^) in the experimental vs. control arm (60 mg/m^2^). A recent meta-analysis on 3,325 subjects reported that adding GO had no impact on remission rate but reduced 5-year cumulative incidence of relapse [OR = 0.81 (*p* = 0.0001)] and improved survival [OR for death = 0.90 (0.82, 0.98), *p* = 0.01] (53). These benefits were confined to those with favorable risk disease (*n* = 246), in whom survival probability at 6 years was 55% without, vs. 76% with GO (odds ratio 0.47, 95% confidence interval 0.31–0.73; *p* = 0.0006) and with intermediate cytogenetics (34% vs. 39%, odds ratio 0.84, 95% CI 0.75–0.95, *p* = 0.005). One of the major adverse effects of GO is that it increases the risk of fatal hepatic injury and sinusoidal obstruction syndrome [formerly known as a veno-occlusive disease (VOD)], especially when administered before HSCT. The AML 17 trial confirmed that single doses higher than 3 mg/m^2^ should not be employed because of increased incidence of VOD and early mortality (54). GO should routinely be combined with 7 + 3 or FLAG-IDA in those with ELN favorable or intermediate risk assuming an acceptable risk of TRM. Fournier et al. also found the addition of GO in patients with mutations in NPM1, CEBPA, FLT3 ITD or TKD, NRAS, KRAS, and SRSF2 to be beneficial (55).

Expression levels of CD33 and the correlation with clinical response have been evaluated from multiple phase 2 and phase 3 clinical trials. The results from two published meta-analyses did not reveal any significant association between leukemic blast CD33 expression and GO clinical efficacy (56, 57). Recently, six nonsynonymous single-nucleotide polymorphisms (SNPs) were reported to have clinical relevance in pediatric patients treated with GO (58). The most interesting of these polymorphisms is CD33 SNP rs12459419 (C<T; Ala14Val), resulting in a shorter isoform of CD33 that lacks exon 2 (loss of the IgV domain) within the CD33 protein (55). Loss of the V-set domain directly impact the binding, internalization, and clinical efficacy of GO is associated with differential response in GO versus no-GO treatment arms. Specifically, patients with at least one copy of the variant T allele (CT/TT genotypes) derived no benefit from the addition of GO (59). Validation studies for the role of CD33 SNP in mediating response to CD33-directed therapy are forthcoming.

## Cpx-351

One-fourth of AML cases is secondary to previous hematological disorders (sAML) or developing after chemotherapy or radiotherapy (tAML) (60). sAMLs and tAMLs are more frequent in older patients, and their prognosis is often worsened by the presence of adverse cytogenetic, high-risk molecular aberrations, and impaired performance status. Allo-HSCT is the only curative therapeutic option in this unfavorable setting, where conventional treatment is usually able to induce less than 40% short-term CRs. In 2017, FDA approved CPX-351 (VYXEOS^®^, Jazz Pharmaceuticals), a liposomal encapsulation of cytarabine and daunorubicin, with a molar ratio of 5:1 for patients with t-AML or with AML and “myelodysplasia related changes” (AML-MRC). The approval followed the phase 2 clinical trial (NCT00788892) of CPX-351 versus 7 + 3 in older patients (60–75 years) with newly diagnosed AML. The study was conducted on 127 patients randomly assigned to receive CPX-351 or 7 + 3. CPX-351 produced a higher CR/Cri rate than 7 + 3 (66.7% vs. 51.2%), without statistical difference in EFS and OS. In a subgroup analysis of patients with secondary AML, the CR/CRi rate was higher (57.6% vs. 31.6%) with an improved EFS (HR = 0.59, *p* = 0.08) and OS (HR = 0.46, *p* = 0.01) (61).

In the setting of newly diagnosed secondary AML, a randomized study conducted on 309 patients, aged 60 to 75 and treated with CPX or 7 + 3 (daunorubicin at the dose of 60 mg/m^2^), showed C/CRi rates of 48% vs. 33% (*p* = 0.02) and event-free survival longer with CPX (*p* = 0.02, medians 2.5 vs. 1.3 months) (62). Recently published data with a median follow-up of 5 years confirm a reduction in the risk of death by 30% (HR 0.7, 95% CI 0.6–0.9, and thus *p* < 0.05, medians 9.3 months CPX, 6.0 months 7 + 3) with the CPX administration (63). The best outcome was observed in patients who promptly underwent transplant after achieving CR.

Given its efficacy, with a favorable safety profile, CPX-351 is now under investigation, alone or in combination, also in the younger population. Few data are available up to now, in a limited number of patients, and thus they should be taken with caution. First, data from a multi-institutional retrospective analysis on 30 younger patients (mean age, 53 years; range, 23–59) with confirmed s-AML showed a worst outcome for CPX-351, with respect to the elderly population. Response rate was unsatisfactory (CR/CRi 27.6%), and median overall survival was shorter (7 months) than reported in the recently published phase 3 trial in patients aged 60–75 years old (64). However, 10/30 patients had TP53 mutation, 19/30 had complex karyotype, and 6 had received prior HMAs, thus representing a very high-risk population with extremely dismal outcome. A phase 2 trial is now prospectively enrolling AML patients with secondary AML aged less than 60 years (NCT04269213), in order to clarify the role of CPX-351 in this setting.

More interest, however, is coming from the possible use of CPX-351 in combination with other drugs, since preclinical data suggest a synergistic activity in combination with venetoclax or FLT3 inhibitors (65). The ongoing V-FAST study (65) is evaluating the safety and establishing the recommended phase 2 dose (RP2D) of CPX-351 combined with other agents for patients with newly diagnosed AML aged 18–75 years. Preliminary data on 26 patients were presented at the EHA 2021 (65). RP2D was established for all the combinations. Even if the results are preliminary, it seems that the combination of venetoclax+CPX-351 is quite toxic (three deaths due to AE, one due to sepsis), with CR rate approximately 50%. On the contrary, the combination of CPX-351 with midostaurin and enasidenib was very well tolerated and able to induce 100% of CR, paving the way for further exploration. The phase 2 of the study is enrolling patients (NCT04075747).

## Azacitidine maintenance

A randomized phase 3 trial (HOVON97) assessed the value of azacitidine as post-remission therapy, in older patients (≥60 years) with AML or myelodysplastic syndrome, in CR/CRi after at least two cycles of intensive chemotherapy. A total of 116 eligible patients were randomly (1:1) assigned to either observation (*N* = 60) or azacitidine maintenance (*N* = 56; 50 mg/m^2^, subcutaneously, days 1–5, every 4 weeks) until relapse, for a maximum of 12 cycles. Fifty-five patients received at least 1 cycle of azacitidine, 46 at least 4 cycles, and 35 at least 12 cycles. DFS was significantly better for the azacitidine treatment group (logrank; *p* = 0.04), and the 12-month DFS was estimated at 64% for the azacitidine group and 42% for the control group. OS did not differ between treatment groups (66).

Also, single treatment with the oral azacitidine formulation CC-486 has been investigated in different clinical settings. In the phase 3, randomized QUAZAR AML-001 trial, CC-486 (300 mg once daily on days 1–14 every 28 days) significantly improved OS and RFS in older AML patients who were in first remission after intensive chemotherapy and not candidates for allogeneic HSCT (67). In a phase 1/2 study in 28 AML/MDS patients in CR after HSCT, CC-486 maintenance therapy showed preliminary efficacy, with an estimated 1-year survival rate of 86% and 81%, respectively (68); the phase 3 trial is ongoing (NCT04173533). In the context of young patients with AML, oral azacitidine should probably find its place in patients unfit for allo-HCT. CC-486 could also represent an excellent bridging therapy to allo-HCT in CR patients waiting for the donor selection; it can be used after consolidation cycles, without the need for hospitalization, thus reducing the risk of relapse.

To date, maintenance therapy with oral azacitidine is licensed only for patients aged ≥55 years with AML and intermediate- or poor-risk cytogenetics who achieved CR/CRi after intensive chemotherapy ± consolidation and are transplant-ineligible.

### HSCT

Upfront allo-HSCT for adult AML patients in CR1 is the gold standard, if cure is the final goal of treatment. Its usefulness was firstly demonstrated in 2005, with a meta-analysis of five studies in which allo-HCT was performed in CR1 AML patients from an HLA-identical sibling donor. This meta-analysis revealed that the efficacy of allo-HSCT for patients with AML in CR1 depends on cytogenetic risk: the beneficial effect of allo-HSCT was evident for the poor-risk group patients, and probably for the intermediate-risk groups, but was absent for the favorable-risk group (69).

Also in 2009, another meta-analysis of clinical trials was performed using a “donor versus no donor” methodology, which showed statistically significant RFS and OS for allo-HCT in young patients with intermediate- and poor-risk AML (70).

The pivotal question in deciding whether to recommend HSCT in CR1 is if the reduction in relapse risk reached with the transplant outweighs NRM. Since the NRM of fit AML patients transplanted with a matched sibling or unrelated donor is approximately 15%, patients with a relapse risk >50% are likely to benefit from allo-HSCT. In recent years, the prognostic classification of AML patients has been refined, and several factors influence the success of intensive chemotherapy. Among these factors, hyperleukocytosis (>100,000/mmc) at diagnosis, secondary AML, adverse karyotype or adverse genetic risk, and a resistant disease have been identified as bad prognostic factors (2). Finally, yet importantly, persistence of MRD positivity after consolidation therapy is also an important independent predictor of relapse risk in AML, and can be used to refine the decision-making process (19). Nonetheless, two scoring systems are able to predict transplant outcome in AML, specifically NRM. The EBMT score comprises age, donor type, HLA disparity, and disease status, and is able to stratify NRM risk between 15% and 45% (71). The HCTCI, derived using a weighted assessment of 17 comorbidities, and the updated comorbidity-age index (age cutoff, 40 years), has also been shown to predict NRM and OS after allo-HCT, and has been validated in AML (72, 73). Recently, a simplified comorbidity index (SCI) has been developed in a single-center cohort of 573 adult patients (217 AML patients) who underwent CD34-selected allo-HCT following myeloablative conditioning. The SCI includes comorbidities associated with a significant increase in NRM: cardiac comorbidity, pulmonary disease, hepatic injury, and renal disfunction. Age with a cutoff of 60 years was also included because it is related to NRM risk (HR 1.64, 95% CI, 1.23–2.19; *p* = 0.001) (74).

Another matter of debate is the optimal conditioning regimen for young, fit patients with AML. In fact, despite optimization of transplant procedures, leukemia recurrence remains the main cause of transplant failure. Recent lines of evidence, coming from randomized clinical trials, demonstrated that reducing the conditioning intensity might not be the right strategy in AML. Recently, a phase 3 clinical trial compared outcomes by conditioning intensity [myeloablative conditioning (MAC) or a reduced-intensity conditioning (RIC)] in adult patients with myeloid malignancy undergoing an allo-HCT while in morphologic CR. Conditioning intensity made no difference in MRD-negative patients who underwent transplantation. In patients with a detectable mutation by NGS, relapse (3-year cumulative incidence, 19% MAC vs. 67% RIC; *p* < 0.001) and survival (3-year OS, 61% MAC vs. 43% RIC; *p* = 0.02) were significantly different between the two arms. Multivariable analysis for NGS-positive patients, adjusting for disease risk and donor group, confirmed the inferior OS for patients receiving RIC compared with MAC (HR, 1.97; 95% CI, 1.17 to 3.30; *p* = 0.01) (75).

The combination of thiotepa, busulfan, and fludarabine (TBF) could represent a valid alternative, as a myeloablative regimen, to busulfan and cyclophosphamide in AML patients. In a registry-based retrospective study reported by the EBMT group (76) and in a multicenter trial (77), TBF regimen confirmed its antileukemic potential with an impressive low number of relapses in patients transplanted in CR1, which translated in a trend towards better LFS in AML patients.

## Conclusions

Intensive chemotherapy, with or without targeted agents, remains the backbone of treatment in fit patients with AML. Treatment of patients with favorable ELN risk does not necessitate an allo-HCT to cure those patients. On the contrary, allo-HCT is the most effective strategy to achieve MRD-negative CR, which is the ideal condition to pursue a long-lasting OS in patients with intermediate or adverse ELN risk. In these patients, it is necessary to further investigate venetoclax with or without intensive chemotherapy or other targeted agents, to evaluate its potential role in increasing MRD-negative CR after induction/consolidation and prior to allo-HCT. Furthermore, maintenance therapy after allo-HCT should be considered for patients with either FLT-3-positive AML or adverse-risk features. Maintenance with sorafenib or midostaurin, even if not formally approved in all countries, demonstrated a survival advantage for patients with FLT-3-positive AML, when administered after allo-HCT, even if burdened with a certain degree of toxicity. Small phase 2 clinical trials suggest that HMAs, with or without drugs targeting bcl-2 or specific molecular markers, could be used as a safe and effective maintenance in patients with adverse ELN risk, with high relapse risk. MRD monitoring, in order to early identify MRD fluctuations or relapse, should be performed in all patients, in order to support an early intervention in those adverse risk patients not receiving any maintenance after allo-HCT. In conclusion, even if the landscape of AML treatment is changing, there is still room for improvement for survival of the fit and young population. Further development of precision medicine, together with the improvement of the knowledge of the biological mechanisms of the interactions between immune cells and AML blasts, will probably lead us to the next level, in order to significantly increase the number of AML patients for whom a curative intent is possible.

## Author contributions

GV, MC, and AI wrote, commented and approved the manuscript. SP and FL wrote the revised version of the manuscript, commented and approved the final version of the manuscript. GV and AI equally contributed to the manuscript. All authors contributed to the article and approved the submitted version.

## Acknowledgments

This study was supported in part by AIL Pesaro Onlus.

## Conflict of interest

The authors declare that the research was conducted in the absence of any commercial or financial relationships that could be construed as a potential conflict of interest.

## Publisher’s note

All claims expressed in this article are solely those of the authors and do not necessarily represent those of their affiliated organizations, or those of the publisher, the editors and the reviewers. Any product that may be evaluated in this article, or claim that may be made by its manufacturer, is not guaranteed or endorsed by the publisher.
